# Vasculogenic mimicry signaling revisited: focus on non-vascular VE-cadherin

**DOI:** 10.1186/s12943-017-0631-x

**Published:** 2017-03-21

**Authors:** Daniel Delgado-Bellido, Santiago Serrano-Saenz, Mónica Fernández-Cortés, F. Javier Oliver

**Affiliations:** 1 IPBLN, CSIC, CIBERONC, Granada, Spain; 2 CABIMER, CSIC, Sevilla, Spain; 3 IPBLN, CSIC, Av. Conocimiento s/n, 18016, Granada, Spain

## Abstract

Vasculogenic mimicry (VM) is a blood supply system independent of endothelial vessels in tumor cells from different origins. It reflects the plasticity of aggressive tumor cells that express vascular cell markers and line tumor vasculature. The presence of VM is associated with a high tumor grade, short survival, invasion and metastasis. Endothelial cells (ECs) express various members of the cadherin superfamily, in particular vascular endothelial (VE-) cadherin, which is the main adhesion receptor of endothelial adherent junctions. Aberrant extra-vascular expression of VE-cadherin has been observed in certain cancer types associated with VM. In this review we focus on non-endothelial VE-cadherin as a prominent factor involved in the acquisition of tubules-like structures by aggressive tumor cells and we summarize the specific signaling pathways, the association with trans-differentiation and stem-like phenotype and the therapeutic opportunities derived from the in-depth knowledge of the peculiarities of the biology of VE-cadherin and other key components of VM.

## Background

Solid tumors require blood vessels for growth, and access to oxygen and nutrients and anti-angiogenic therapies are designed to target vascular ECs to form tumor blood vessels. Whereas numerous preclinical models have recognized the efficient use of angiogenesis inhibitors to limit tumor growth, collectively only a growth delay has been achieved in the clinic [1]. This is in part due to the fact that tumor vasculature is more complex than expected and alternative mechanisms for re-vascularization might be taking place. A large number of studies in pathology have described a high degree of plasticity associated with aggressive cancer. In 1999, Maniotis et al. [2] presented a new interpretation of previous findings describing cancer cells covering non-endothelial vascular channels that contained red blood cells. This was the initial report defining tumor cell VM as the de novo formation of perfusable, matrix rich, vasculogenic-like network in 3D matrix in vitro, which resembled the matrix-rich network observed in aggressive tumors in patients [3]. The initial morphological, clinical and molecular characterization of VM was performed using human melanoma as a model. In addition to melanoma, vasculogenic mimicry (VM) has also been characterized in carcinomas of lung, prostate, bladder, kidney, ovary and breast, sarcomas and gliomas. Kaplan-Meier survival analyses indicated that patients with VM in their tumors have a poor clinical outcome compared with patients with tumors that do not exhibit VM. Table 1 shows the main differences at molecular level between blood vessels and VM networks.

**Table 1:** Differences in tumor-VM and ECs-dependent angiogenesis and VM inducer and suppressor molecules

Normal endothelial cells	Vasculogenic mimicry cells
Similarities	
VE-cadherin positive	
E-selectin positive	
CD34 positive	
Differences	
TIE-2 positive	TIE-2 negative
VEGFR-1, 2 positive	VEGFR-1, 2 negative
P-selectin positive	P-selectin negative
VCAM-1/CD106 positive	VCAM-1/CD106 negative
CD31/PECAM-1 positive	CD31/PECAM-1 negative *13 (Subpopulations PECAM-1 positive melanoma cells)
TIE-1 negative	TIE-1 positive
VEGF-C negative	VEGF-C positive
Neuropilin 1 negative	Neuropilin 1 positive
Endoglin negative	Endoglin positive
Tissue factor pathway inhibitor 1 (TFPI1) negative	Tissue factor pathway inhibitor 1 (TFPI1) positive
Laminin 5 gamma 2 chain (LAMC2) negative	Laminin 5 gamma 2 chain (LAMC2) positive
EphA-2 negative	EphA-2 positive

The distinctive pattern of VM networks appears to recapitulate embryonic vasculogenesis patterns and this resemblance suggests that aggressive tumor cells convert to an undifferentiated, embryonic-like phenotype. Gene expression analysis demonstrated that aggressive melanomas capable of VM express genes associated with multiple cellular phenotypes, including characteristics of epithelial cells, endothelial cells and fibroblasts [4, 5]. However, the different molecular mechanisms that generate VM are still unclear. One of the most conspicuous molecular determinants in the acquisition of VM capabilities is the expression of the endothelial cell marker VE-cadherin. In the current review, we focus on the connection between VE-cadherin and its consequences in the gain of the VM phenotype which is also associated with cell plasticity and trans-differentiation of cancer stem cells present in VM.

## VE-cadherin in VM

**Fig. 1:**
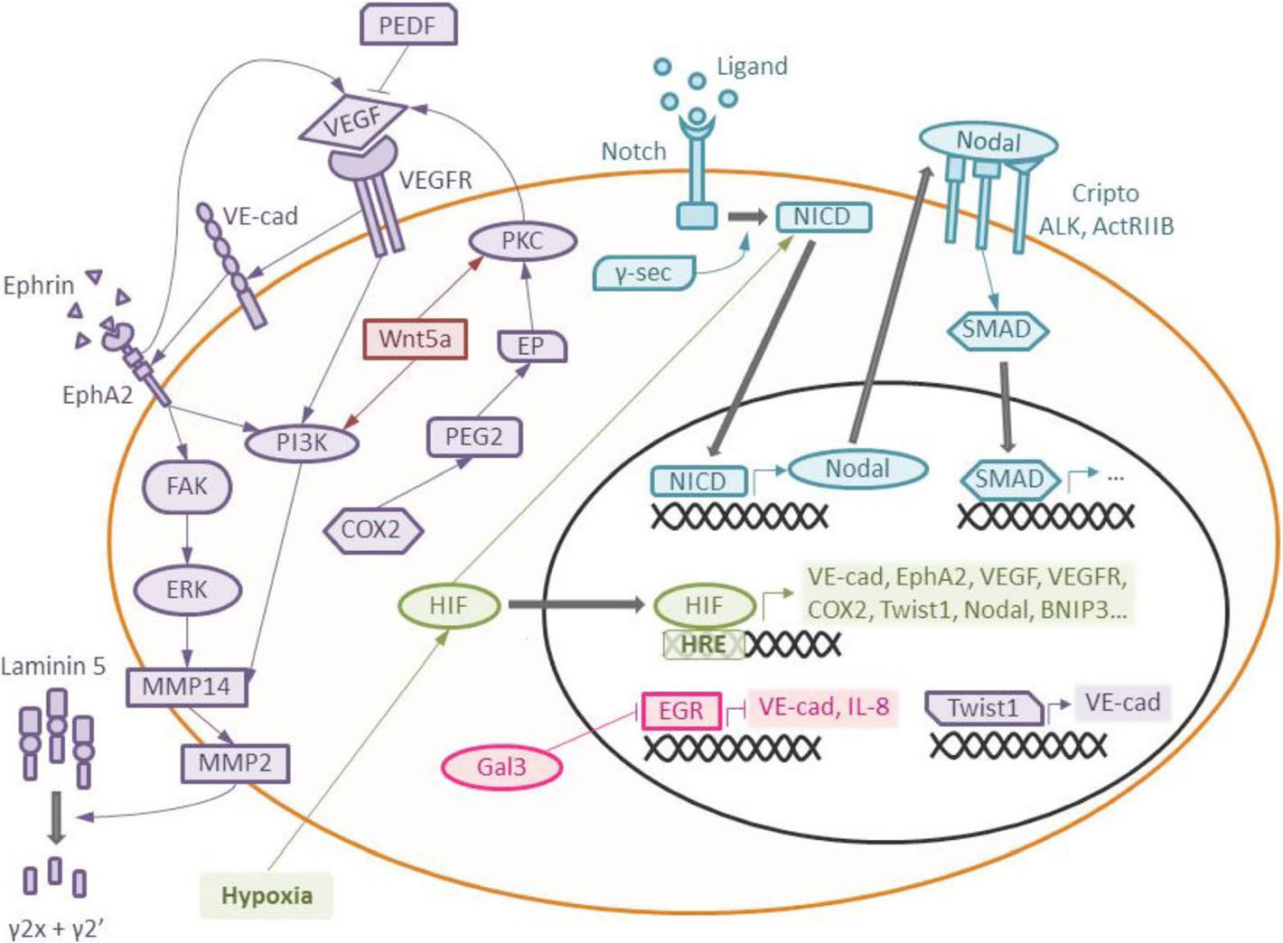
Main signaling pathways involved in vasculogenic mimicry. In vascular signaling (*purple*), VE-cadherin, EphA2 and VEGF lead to proteolytic cleavage of laminin 5 and release of pro-migratory γ2x and γ2’ fragments in the extracellular matrix. Galectin 3 supports the vascular pathway, since it enhances the expression of VE-cadherin. Stem cell signaling (*blue*), controlled by Notch and Nodal, up-regulates genes for pluripotency and de-differentiation. Hypoxia (*green*) contributes to all previous pathways by mediating expression of some crucial signaling molecules. Finally, Wnt proteins may promote vasculogenic mimicry through the activation of PKC and PI3K signaling, though it could play a role in tumor suppression in certain cases

VE-cadherin, Notch or hypoxia-inducible factor 1-α (HIF1-α) are among the most relevant signaling molecules involved in the three leading pathways that control VM: vascular, hypoxia and embryonic/stem cell signaling pathways. All these pathways are complex and interconnected, with a large number of different molecules performing together and modulating the outcome effect in a different way (reviewed in [6–9]) (Fig. 1).

VE-cadherin is a trans-membrane protein commonly expressed in endothelium, where it is responsible for cell-cell adhesion [10]. Although VE-cadherin used to be considered specific for ECs, its expression has been strongly associated with aggressiveness and VM in melanoma. Surprisingly, VE-cadherin can be found in highly aggressive tumor cells but not in non-aggressive ones. Moreover, its down-regulation in melanoma implied the loss of VM formation [11].

VE-cadherin is the best known cadherin in the context of vascular adhesion but the insights of its role in VM in aggressive tumor cells are only beginning to be well understood. The fact that VE-cadherin is key to understand VM was discovered by Hendrix and col in 2001 [12]. In this seminal study, they showed that VE-cadherin is expressed in aggressive melanoma cells while its knockdown prevented VM. On the other hand, in vivo experimental models also demonstrated that mice deficient in VE-cadherin die by severe vascular defects [13]. The reason for the expression of VE-cadherin in non-endothelial cells is mostly unclear, but in hepatocellular carcinoma (HCC) it may be due the to nuclear localization of Twist1. This transcription factor has been shown to bind VE-cadherin promoter, enhancing its activity [14]. In melanoma, VE-cadherin expression has been related with the activation of the Nodal/Notch pathway [15, 16] and hypoxia-inducible factors [17, 18]. Finally, it has been suggested that human epidermal growth factor receptor 2 (HER2) up-regulates VE-cadherin in breast cancer cells [19].

**Fig. 2:**
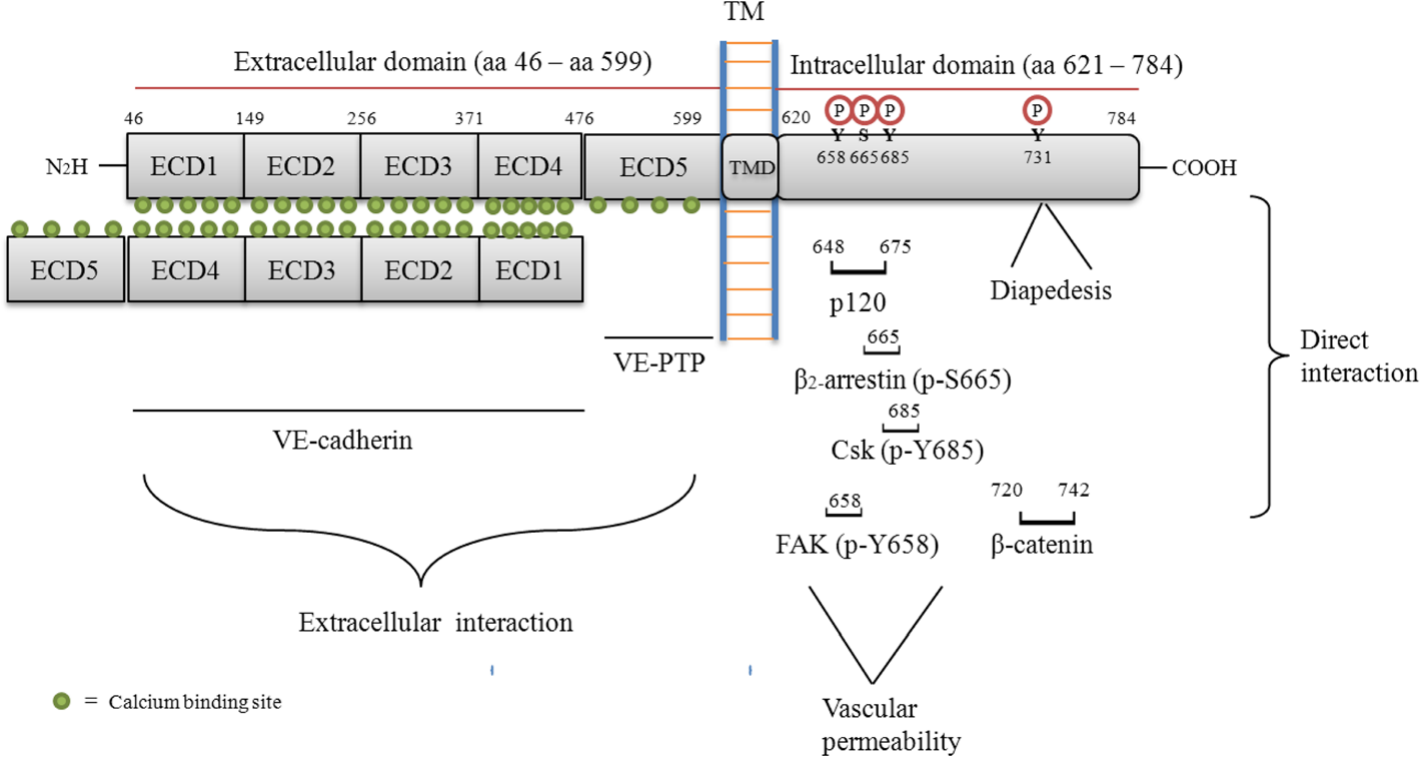
Structural features of VE-cadherin and implication in signalling. Further details are given in the text for the VE-cadherin subheading

Structurally, VE-cadherin has five extracellular calcium domains (ECD:aa46-aa599) that can form cis-homodimers with other VE-cadherin or similar dimers in trans- through ECDI-ECDIV, that present on adjoining cells to support cell-to-cell recognition and adhesion. ECDV is required for binding to VE-PTP. VE-cadherin also has a trans-membrane domain (TMD: aa600-aa620) and an intracellular domain involved in post-translational modifications (PTM) (Fig. 2). In fact, 13 possible residues of VE-cadherin that can be phosphorylated in humans (Uniprot KB/Phosphosite Plus) have been described but the most relevant ones, and also the most studied, are residues Y658, S665, Y685 and Y731. Phosphorylation in S665 of VE-cadherin takes place through serine/threonine kinase P21 protein (Cdc42/Rac)-activated kinase 1 (PAK) in response to VEGF that also promotes clathrin-dependent internalization of VE-cadherin [20]. Recent studies have shown that Y658 residue is a target of focal adhesion kinase (FAK) in tumor-associated endothelial cells, and identify FAK as a key regulator of endothelial cell barrier function controlling tumor metastasis [21]. Tyrosine phosphorylation of VE-cadherin Y685 causes its internalization mediated by Src, thus playing an important role in in vivo vascular permeability [22, 23]. In contrast, mice expressing Y731F VE-cadherin mutant display deficient neutrophil-extravasation; indeed, phosphorylation of Y731 is induced through inflammatory mediators, such as histamine, that promote neutrophil-extravasation [23] (see Fig. 2).

Vascular endothelial growth factor A (VEGF-A) is known to contribute to endothelial cell proliferation, including tumor-induced angiogenesis, and has been associated to VM in ovarian carcinoma and melanoma [8]. In fact, in ovarian cancer cells VEGF-A may up-regulate most of the signaling molecules in the VE-cadherin signaling cascade: VE-cadherin itself, Epithelial cell kinase (EphA2) and MMP-2 [7]. We will analyze below different key pathways and molecules involved in VM and their connection with VE-cadherin.

## VE-cadherin-dependent and independent signaling in VM

### Vascular endothelial growth factor (VEGF) signaling

When we refer to VE-cadherin it is necessary to describe VEGF receptors (VEGFRs) because they represent the most important pathway to regulate VE-cadherin function and turn-over. VEGFR-2 (flk-1/KDR) and VEGFR-1 (FLT-1) are the most important receptors in this pathway although at least five different VEGF isoforms are also known in human. These receptors are stimulated by vascular endothelial growth factor (VEGF-A) and represent a crucial regulatory system of endothelial growth in a normal cell physiology context; in contrast VEGF is also an endothelial-specific mitogen and potent activator of vascular permeability secreted by tumor cells [24]. VEGFR-2 is highly expressed in vascular ECs thus forming the primitive tubular vessels called vasculogenesis. In contrast, VEGFR-1 is also highly expressed in tumor cells with capacity to form VM; this is the case of malignant melanoma [25]. As mentioned above, VEGF-A promotes vascular permeability by weakening adherent junctions and tight junctions, resulting in transient opening of the endothelial cell-cell contacts [26]. In fact, VEGF-A promotes tyrosine phosphorylation of VE-cadherin and its binding to the partner β-catenin, plakoglobin and p120, via Src-dependent mechanism [27]. VE-cadherin is inhibited following its phosphorylation in mice deficient in Src [27]. VE-cadherin may also be associated with and inhibit VEGFR-2 phosphorylation and subsequent internalization [28]. This association promotes the phosphorylation of components of the adherent junctions by Src, thus impairing the integrity of the endothelial barrier and promoting tumor cell extravasation and diffusion in pathological models [29]. In addition, VEGF-A mediates phosphorylation of VE-cadherin internalization through sequential activation of Src, the nucleotide exchange factor of Vav2, Rho GTPase Rac, leading to a downstream effect on the serine/threonine kinase P21 protein (Cdc42/Rac)-activated kinase 1 (PAK). Finally, phosphorylated PAK phosphorylates VE-cadherin, triggering their internalization [26]. Moreover, VEGF signaling reduces the association between VE-cadherin and p120-catenin promoting clathrin-dependent VE-cadherin endocytosis [30]; binding of p120 to VE-cadherin prevents its internalization, while p120 silencing leads to degradation of VE-cadherin, and loss of cell-cell contacts [30].

VEGF binding to VEGFR-1 can enhance other variety of signaling pathways. Activation of tyrosine kinase Src and extracellular regulatory kinases 1 and 2 (ERK1/2) leads to cancer cell invasion and migration. In melanoma, VEGFR-1 seems to promote VM via PI3K/PKC pathway. VEGFR-1 also mediates angiogenesis through activation of PI3K/Akt pathway [7, 8]. Apart from this, VEGFR-1 is necessary for the expression of VE-cadherin [25]. As for VEGFR-3, it has been proved in melanoma that endothelin-1 (ET-1) can enhance its expression and also the expression of its ligands VEGF-C and -D. The signaling pathway involves ET-1 binding to its receptor ETBR, which activates hypoxia-inducible factors. The binding also leads to phosphorylation of VEGFR-3 (probably mediated by Src and β-arrestin-1), which becomes able to trigger the activation of MAPK signaling cascade. All of these events lead finally to the promotion of cell migration and VM [31].

Different evidences support the fact that VEGF expression is determined by EphA2 in breast cancer and pancreatic islet carcinoma cells. Cyclooxygenase-2 (COX-2) also stimulates the expression of VEGF in other tumor cell lines [7–9].

Cyclooxygenase-2 catalyzes the reaction that converts arachidonic acid into primarily prostaglandin E2 (PGE2). This molecule binds to prostanoid receptors (EP1-4) that activate protein kinase C (PKC) and epidermal growth factor receptor (EGFR) signaling pathways. Both of them lead to decreased apoptosis and increased tumor proliferation, invasion and angiogenesis. In particular, PKC signaling up-regulates VEGF expression [7–9]. In breast cancer, elevated COX-2 expression results in the increased ability to form VM networks, while its knockdown leads to a reduced VM. Importantly, VM capability of breast cancer cells with low COX-2 can be restored if PGE2 is added to the culture [32]. It has been reported that EP3, but not EP4, modulates VM in breast cancer [33].

On the contrary, VEGF/VEGFR-1 signaling can be inhibited by the pigment epithelium-derived factor (PEDF), a glycoprotein that belongs to the family of serine protease inhibitors. Furthermore, PEDF induces tumor cell differentiation and apoptosis, yet probably prevents VM development, since it is usually down-regulated in aggressive tumor cells. Even more, PEDF silencing favors VM in melanoma [7, 8].

Tissue factor (TF) and TF pathway inhibitors (TFPI-1/2) are involved in VM as well. Specifically, knockdown of TFPI2 inhibited MMP-2 activity, suggesting a role for TFPI2 in extracellular matrix remodeling associated with VM channel formation [7, 8].

Finally, an opposite role of VEGF signaling in VM has been proposed too. All the evidence presented so far indicates that VEGF promotes VM, but it has been suggested that, on the contrary, VM might increase in the absence of this signaling pathway: VEGF would promote angiogenesis, while VEGF blockade could enhance some other strategies for tumor cell survival, including VM [34].

### VE-cadherin and EphA2 in VM

EphA2 has also been shown to be related to VM. EphA2 is a protein tyrosine kinase whose phosphorylation and activity depend on the binding of ephrin-A1, although it has been reported that EphA2 can also be constitutively active in some tumor cells [35]. As VE-cadherin, EphA2 was found to be expressed only in highly aggressive tumors, where it was tyrosine-phosphorylated. When cells were cultured on a three-dimensional matrix and labeled with anti-phosphotyrosine antibodies, the staining showed that tyrosine-phosphorylation was present mainly in the areas of tubular network formation. General inhibitors of protein tyrosine kinases as well as specific silencing of EphA2 hindered the development of vascular networks, suggesting a potential role for phosphorylated EphA2 in this process [35]. In VM, VE-cadherin and EphA2 co-localize at the plasma membrane, specifically in cell-to-cell contact regions. Knockdown of VE-cadherin resulted in a reorganization of EphA2 location, which seemed to move into the cytoplasm. Moreover, there was a decrease in EphA2 phosphorylation. On account of these results, it seems that VE-cadherin may help EphA2 translocate to the plasma membrane, [36, 37].

PI3K up-regulates both the activity and expression of matrix metalloproteinase-14 (MMP-14) in highly aggressive cells. MMP-14 in turn activates MMP-2, and finally cleaves laminin 5γ2 chain to produce the γ2’ and γ2x fragments, which are secreted to the extracellular matrix to promote migration in various tumor cell types, like breast, colon carcinomas and hepatoma [38]. More precisely, they activate the secretion of γ2’ and γ2x pro-migratory fragments leading to VM in melanoma, gallbladder and ovarian carcinomas [39–41]. Furthermore, poorly aggressive melanoma cells (which cannot normally engage in VM) could form vasculogenic-like networks when seeded on collagen gels that had been pre-conditioned by highly aggressive melanoma cells. Aggressive cells were removed before apparent formation of tubular networks, but the examination of the a-cellular matrices showed the presence of laminin-positive patterned networks. If these matrices where treated with antibodies anti-laminin-5γ2 chain before seeding the poorly aggressive melanoma cells, they could no longer develop tubular networks. Altogether, these results confer great importance to this signaling cascade and laminin-5γ2 in particular.

As for FAK, over-expression of EphA2 caused an increase of MMP-2 in a way dependent on FAK [42]. FAK itself is highly phosphorylated at positions Y397 and Y576 in highly aggressive melanoma, but not in poorly aggressive melanoma cells; this post-translational changes are indicative of a fully active FAK. FAK-related non-kinase (FRNK) can interact with focal adhesion proteins but it lacks kinase activity, so it is considered a dominant-negative FAK protein. Expression of FRNK in aggressive melanoma decreased invasiveness, migration and ability to form tubular networks on collagen gels. It also reduced the levels of phosphorylated ERK1/2, whose inhibition decreased the levels of urokinase as well as the activity of MMP-14 and MMP-2. These results hint at a signaling cascade where EphA2 promotes VM via FAK and ERK, meeting the PI3K pathway at MMP-14 and leading henceforth to the cleavage of laminin-5γ2 [43].

### Notch/Nodal

Nodal/Notch belongs to the superfamily of the transforming growth factor β (TGF-β). It is essential during embryonic development, since it maintains undifferentiated status of embryonic stem cells in order to ensure growth and development. Notch is a trans-membrane receptor with four different isoforms (Notch1-4), all of which undergo cleavage by γ-secretase after binding of one out of five possible ligands: Delta-like-1/3/4 and Jagged1/2. Notch intracellular domain (NICD) is released into the cytoplasm and it is imported to the nucleus, where it regulates gene expression, including expression of Nodal. Notch signaling pathway is involved in differentiation, although cell fate depends on cellular context. Also Notch is associated with the development of vascular networks in embryonic stages, so it may have a relationship with VM in cancer. Nodal binds several membrane receptors (Cripto-1, activing-like kinase receptors (ALK4/7), ActRIIB, and/or TGF-β receptor I and II), causing phosphorylation of SMAD2/3, enabling its association with SMAD4 and their translocation to the nucleus to activate the expression of Nodal target genes, including LEFTY, which is responsible of negative feedback in this signaling pathway, as it directly inhibits Nodal.

It has been reported that disruption of the Notch signaling cascade with γ-secretase inhibitors stabilizes VM networks in melanoma, that is, Notch might have an attenuating effect in VM [44]. Nevertheless, there are evidences supporting a positive relationship between Notch and VM: Notch-4 was shown to be highly expressed in several aggressive melanoma cell lines capable of VM, where it seemed to play a crucial role in the up-regulation of Nodal expression as well. Furthermore, antibody-mediated blocking of Notch signaling resulted in an impairment of VM ability, which could be restored with the addition of Nodal [45]. Nodal expression normally stops after cell differentiation during human development, but it can be rescued in tumor cells, for instance, melanoma and breast cancer cells. This pathway is related to tumor progression, aggressiveness, VM and VE-cadherin expression in aggressive melanoma cells [15, 16].

### Hypoxia

Oxygen depletion is a common situation in growing tumors; therefore tumor cell survival and, consequently, malignancy are often dependent on their adaptation to hypoxia. Hypoxia-inducible factors (HIFs) and hypoxia responsive elements (HRE) play a crucial role in this context; HIFs are transcription factors stable in hypoxic conditions, while HRE are the genomic regions where HIF bind to mediate gene expression. These transcription factors are stabilized during conditions of hypoxia and they are critical to induce genes for tumor cell adaption to a hostile and changing microenvironment.

Notably, some potential hypoxia target genes containing HRE are involved in VM, such as VEGF-A, VEGFR-1, EphA2, Twist, Nodal, COX-2 and VE-cadherin [8, 46]. The later has been found to contain up to six HRE upstream of the promoter [6]. As a result, hypoxia has been reported to promote VM in a wide variety of tumor cell lines [47–49]. In murine models of melanoma, VM development in conditions of ischemia was significantly increased. Moreover, there is a positive relationship between expression of HIF-1α and VEGF in ischemic tumor cells [50]. Instead of VEGF, in human fibrosarcoma, HIF-1α promoted VM by up-regulating Neuropilin-1 (NRP-1), a VEGF receptor and co-receptor of VEGFR-2. Unexpectedly, silencing of NRP-1 completely disrupted tumor formation in fibrosarcoma [51].

In human melanoma, hypoxia induced over-expression of anti-apoptotic protein B-cell lymphoma 2 (Bcl-2), which in turn increased VE-cadherin expression [17]. VE-cadherin was also up-regulated by HIF-1α in esophageal carcinoma [18]. To summarize, hypoxia is an essential trigger for vascular signaling pathways in the process of vasculogenic mimicry. Hypoxia may also influence VM through BNIP3, a protein that belongs to the family of Bcl-2. Expression of BNIP3 is remarkably up-regulated under hypoxia, allowing its contribution to cell migration and VM development in melanoma. BNIP3 enhanced these processes by modulating the organization of the actin cytoskeleton while BNIP3 knockdown completely inhibits VM, changed cell size and shape, driving to formation of actin stress fibers, and reduced tight and adherents junctions [52].

In glioblastoma, HIF-1α expression is mediated by mammalian target of rapamycin (mTOR). Specific inhibition or silencing of mTOR disrupted VM formation, especially under hypoxia but also in normoxic conditions, since some VM signaling molecules (HIF-1α, MMP-2 and MMP-14) were down-regulated [47]. In 2007, A. Le Bras and col [53] showed that the expression of VE-cadherin is controlled and regulated by a basal, non-endothelial specific promoter, containing six putative hypoxia responsive elements (HRE) which are binding sites for HIF-1α and HIF-2α. Consistent with this, HIF-2α (but not HIF-1α) regulated the expression of VE-cadherin in hypoxia as well as in normoxia. HIF-1α also induced the expression of VE-cadherin and modulated VM in esophageal carcinoma cells [18]. Following knockdown of HIF-1αVM in inhibited and the expression of VM-related genes reduced, for example EphA2, VE-cadherin or laminin-5γ2 but not MMP-2 in vitro and also in vivo [18]. Finally, it should be noted that mediating gene expression is not the only way by which hypoxia can influence VM: HIF-1α is known to stabilize NICD [54], whose role was explained above.

### Galectin-3

Galectins are carbohydrate-binding proteins whose functions include cell adhesion and migration [9]. Galectin-3 (Gal-3) was proven to have oncogenic and angiogenic properties, being up-regulated, for example, in metastatic melanoma and breast cancer cells. Gal-3 silencing reduced invasiveness and inhibited VM formation in melanoma through down-regulation of the expression of some endothelial markers, such as VE-cadherin, together with the release of interleukin-8 (IL-8) was found to decrease. IL-8 is pro-angiogenic interleukin which modulates the expression of MMP-2, whose important role in VM was mentioned above [55]. Gal-3 mediates gene expression by inhibiting early growth response protein 1 (EGR-1), which loses its ability to bind (and thereby to repress) VE-cadherin and IL-8 promoter regions [55]. Therefore, the presence of Gal-3 allows the transcription of EGR-1 target genes. Consistent with this, gene expression microarrays analysis after silencing Gal-3 showed that Gal-3 regulates the expression of multiple genes, and has a negative influence on endothelial markers aberrantly expressed in highly aggressive melanoma cells such as VE-cadherin, IL-8, fibronectin-1, endothelial differentiation sphingolipid G-protein receptor-1 and MMP-2 [56]. It has been shown that shRNA of Gal-3 decreased VE-cadherin, and IL-8 promoter activity due to enhanced transcription of factor early growth response-1 (EGR-1) [57].

### Wnt family

The Wingless (Wnt) family proteins is related to a large range of physiological processes, including embryonic patterning, cell proliferation, migration, cell differentiation. It plays an important role in endothelial cell differentiation, vascular development and angiogenesis [58], especially Wnt5a, a member of the non-canonical Wnt signaling. However, the role of Wnt5a in cancer is still being debated: in certain settings has been described as a tumor suppressor, but it is generally involved as a pro-metastatic factor [59].

Wnt5a is known to promote cell migration by modulating several proteins of the cytoskeleton, providing tumor cells with abilities in relocation. Moreover, Wnt5a releases intracellular calcium, activating calcium-dependent proteins such as protein kinase C (PKC), which is essential for invasion in cancer cells. All these properties, among others, suggest that Wnt5a is a cancer-promoting molecule [60]. PKCα has been shown to be involved with Wnt5a in EMT and VM in ovarian cancer cells, where the expression of Wnt5a and PKCα were correlated and PI3K levels were enhanced upon up-regulation of Wnt5a [61]. Over-expression of Wnt5a mediates VM formation in ovarian cancer and lung cancer [62, 63]. In 2015, Lisha Qi et al. [62] reported that Wnt3a expression in HT29 colon cancer cells promoted the capacity to form tube-like structures and increased the levels of proteins involved in VM such as VEGFR-2 and VE-cadherin. In addition, the antagonist Dickkopf-1(Dkk-1) reverted the capacity to form VM and decreased the expression of VEGFR2 and VE-cadherin in Wnt3a-overexpressing cells.

On the other hand, Wnt5a may perform as a tumor suppressor in certain cancers by inhibiting β-catenin-mediated transcription via several different molecules, preventing the expression of potential oncogenes. For this reason, the opposing effects of Wnt proteins remain controversial [60].

### Epithelial Mesenchymal Transition (EMT) in VM

EMT is a dynamic biological process where polarized epithelial cells lose their epithelial properties and gain typical characteristics of mesenchymal cells [64]. The best known regulator of EMT is Transforming Growth Factor-beta (TGF-β), whose role in EMT is well-established [64, 65]. In 2008, Myriam Labelle and col [66] showed that VE-cadherin is induced in EMT in mammary tumor cells and it is also aberrantly expressed in invasive human breast carcinomas. In addition, VE-cadherin influenced the levels of SMAD2 phosphorylation and the expression of TGF-target genes. Thus, VE-cadherin might promote tumor progression by contributing to tumor angiogenesis as well as by enhancing tumor cell proliferation via TGF-β signaling. Recently, it has been reported that Zinc finger E-box binding homeobox 2 (ZEB2) fosters VM by TGF-β induction of EMT in HCC where ZEB2 over-expression significantly enhanced cell mobility and VM formation. Up-regulation of ZEB2 increased VE-cadherin, VEFGR-2 and VEGFR-1 expression as well as MMP-2 and MMP-9 [67]. ZEB1 down-regulation decreased the expression of VE-cadherin and VEGFR-2 in colorectal carcinoma, which are characteristic of ECs. In conclusion, ZEB1 promote VM formation by inducing EMT in colorectal carcinoma through the inhibition of VE-cadherin.

Other protein that has been identified to have a prominent role in EMT is Twist1. This protein binds DNA using similar E-box sequence motifs repressing E-cadherin and up-regulating mesenchymal markers expression; over-expression of Twist1 significantly enhanced cell mobility, invasiveness and promoted VM formation in HepG2 cells while chromatin immunoprecipitation showed that Twist1 binds to the VE-cadherin promoter and enhances its activity [68].

### Cyclic adenosine monophosphate (cAMP)

cAMP is an essential second messenger involved in a number of cellular processes, such as cell growth and differentiation. It has also been linked to VM in cancer [69, 70]. Firstly, a rise in cAMP levels reduced VM in cutaneous and uveal melanoma. This inhibition appeared to be dependent mostly on the exchange protein directly activated by cAMP (Epac), but not on protein kinase A. Secondly, VM impairment was associated with an inhibition of ERK and PI3K/Akt signaling [70, 71], both of which were previously mentioned as important participants in the vascular pathways.

Apart from vascular signaling, cAMP may be involved in Notch signaling during endothelium development, so there might be an association with VM, too. The exact relationship between cAMP and Notch in endothelial cell differentiation remains unknown, but cAMP has been shown to modulate presenilin-1 (a component of γ-secretase) in neurons [72].

Besides the different signaling described above other key elements regulating VM are presented in Table 2.

**Table 2:** VM inducer and suppressor molecules

VM inducers	References
HIF1α	[50]
Twist1	[14]
Nodal	[45]
Wnt5a	[61]
VE-cadherin	[111]
EphA2	[35]
Laminin 5γ2	[39]
CD133	[116]
VEGFR-1/2/3	[25]
	[31]
	[118]
PECAM1	[133]
Desmoglein 2	[134]
VM suppressors	
FAK-related nonkinase	[133]
PEDF	[135]
cAMP	[71]
TIMP-2	[136]
AP-2α	[133]
miR-26b (EphA2)	[137]
miR-200a (EphA2)	[138]
miR-1236 (PI3K)	[139]
miR-27a-3p (VE-cadherin)	[140]
miR186 (Twist1)	[141]
hsa-mir-299–5p	[142]
miR-409-3p	[143]
miR-124	[144]
	[145]
miR-Let-7f	[146]

## Vasculogenic mimicry and tumor microenvironment

For a long time, it has been assumed that tumor microenvironment (TME) plays an important role in development and progression of tumors, including invasion and metastasis [73, 74]. This physico-chemical niche includes stromal cells and fibroblasts, blood vessels and oxygen availability, immune cells, extracellular matrix (ECM), and cytokines [75]. Both, TME and tumor, present a bidirectional interaction that regulates several processes in tumor progression. One if these regulated processes is VM.

The first evidence of the influence of TME in VM was proposed by Hendrix et al. in 2002 [76]. They induced ischemic environment by surgery of the femoral artery in nude mice and inoculated highly and poorly aggressive human melanoma cells. They observed that highly aggressive cells, but not poorly aggressive, overlapped with endothelial cells in vasculogenesis of ischemic muscle. They also found by immunohistochemistry that only highly aggressive melanoma cells, but not control cells, strongly expressed Notch-3 and Notch-4 [76]. In a different study, they demonstrated that aggressive melanoma cells can modify the ECM and reprogram the poorly aggressive ones and inducing VM. Microarray analysis confirmed differential gene expression in poorly aggressive melanoma cells preconditioned with microenvironment of aggressive melanoma cells. As mentioned before, among up-regulated genes Eph2A, VE-Cadherin, TIE-1, VEGF-C, metalloproteases (MMP) and γ2 chain of laminin 5 (Ln5γ2) were present [77]. Cooperation between endothelial cells and tumor cells in VM formation has also been demonstrated in lung cancer [78]. Moreover, aggressive melanoma cells but not poorly aggressive melanoma cells induce VM in normal human mesenchymal stromal cells in co-culture. This effect is mediated by VEGFA [79]. Treatment against aggressive melanoma cells may ignore changes in ECM, decreasing the effectiveness of the therapy.

Relationship between cancer-associated fibroblast (CAFs) and VM has also been demonstrated. Conditioned medium of CAFs induces VM in hepatic tumor cells in vitro, and co-implantation of CAFs and hepatic tumor in mouse xenografts showed an increase in VM formation [80]. Additionally, CAFs were present among tumor cells involved in VM formation. This effect is mediated by paracrine effect of TGF-β and SDF1, which promote VE-cadherin, MMP-2 and LAMC2 expression [80]. A recent study demonstrates that TGF-β1 plays an essential role to recruit bone marrow-derived mesenchymal stem cells (MSC) to the tumor. TGF-β1 is involved in MSC differentiation to CAFs which indeed develop VM. Besides, MSC showed tropism with M2 macrophages, that have been related to tumor aggressiveness [81].

Macrophages drill channels and promote microcirculation in ischemic heart [82]. In a tumor context, macrophages have capacity of produce tubular network and they have been also identified in VM formation. These macrophages connect to blood vessels and express EMC degrading proteases [83]. High number of infiltrated M2 macrophages have also been observed in GBM tumor samples. These tumor associated macrophages induce COX-2 dependent VM and their effect is inhibited by Celecoxib [84].

Otherwise, conditioned medium from co-culture of umbilical vein endothelial cells (HUVEC) and HTR-8 trophoblast cells in a three-dimensional system induces VM in ovarian tumor cells (OVCAR-3). These results show that chorionic gonadotropin (HCG) is essential in this induction. HCG enhances HIF-1α and vascular markers. Anti-HCG antibodies reverse this effect. However, siRNA of HCG in OVCAR-3 do not inhibit VM, demonstrating that this induction is due to microenvironment [85].

## CSCs in vasculogenic mimicry

VM requires an adaptive response of tumor cells and cellular “plasticity” is an essential property for this purpose. Cancer Stem Cells (CSCs) are by definition the tumor cell subpopulation with highest plasticity. In this context, “plasticity” is defined as the capability of pluripotent CSCs for trans-differentiation.

In 1994, Lapidot et al. and Caceres-Cortes et al. reported the first evidence of cancer initiating stem-like cells by injection of different leukaemia cell subpopulations in mice [86, 87]. Today CSCs have been determined in a wide range of solid tumors like breast [88], gliomas [89], prostate [90], melanoma [91, 92], lung [93, 94], colon [95–97], pancreatic [98], head and neck squamous cell [99], liver [100], non-melanoma skin cancer [101] and renal carcinoma [102].

CSCs represent an aggressive subpopulation of tumor cells with capacity of self-renewal, multi-lineage differentiation (stemness), tumor initiation and resistance of radio- and chemotherapy [89, 103, 104]. CSCs phenotype is enhanced in perivascular niche of several tumors and this correlates with aggressiveness [105, 106]. Furthermore, CSCs may trans-differentiate to ECs. This fact demonstrates the implication of CSCs in vascularization [107]. Also, several works have linked CSCs with VM capacity in different tumor types. In triple-negative breast cancer (TNBC), VM capacity statistically correlates with CD133 CSCs marker expression [108], and this co-relation was also demonstrated in other tumors like uveal and cutaneous melanoma, gliomas, hepatocellular carcinoma (HCC), non-small cell lung cancer (NSCLC) [108–110].

To have a global view of the importance of VM and its interaction with CSCs we summarized above some key findings linking these two aspects in different tumor types:

### Melanoma

Aggressive melanoma produces VM and it is inhibited by down-regulation of VE-cadherin [111]. Melanoma cancer stem cells (MCSCs) are identified by the expression of CD133 and ABCB5. These cells express VE-cadherin and they are located in the perivascular niche and implicated in VM [112]. MCSCs also express VEGFR-1 and are required for VE-cadherin-dependent VM and tumor growth [25]. Anti-VEGF treatments have been proposed in the treatment of melanoma although with low efficiency to target abnormal tumor angiogenesis, due to the acquisition of anti-VEGF by VM induction. This process is mediated by HIF-1α and involves MCSC in perivascular niche [34]. It has also been reported that PECAM1+/VEGFR-2– subpopulation of melanoma cells induces VM in a PECAM1-dependent process. This process is also dependent on AP-2α, a transcription factor that represses PECAM1 expression: while knockdown of AP-2α up-regulates PECAM1 and promotes tube formation, lentiviral re-introduction of AP-2α down-regulates PECAM1 and inhibits tube formation [113].

### Gliomas

VM is proposed as an important target in glioblastoma because abnormal vasculature induces the loss of blood–brain barrier and contributes to brain edema formation. The expression of VE-cadherin in glioblastoma stem-like cells (GSLCs) has been reported [114]. Based on this premise, Mao et al. demonstrated that VE-cadherin is up-regulated under hypoxic conditions in a way dependent on HIF-1α and HIF-2α and contributes to hypoxia-induced VM [115]. In addition, GSLCs express vascular endothelial growth factor receptor-2 (VEGFR-2) as well as other VM markers. shRNA for VEGFR-2 inhibits tube-like structures formation, VM and vascularization, but also self-renewal and tumor xenograft initiation. For this reason, VEGFR-2 has been proposed as an essential component for the maintenance of stemness capacity and vascularization. VEGFR-2 is necessary for the trans-differentiation of GCSCs to mural cells [116–118]. Other stem-like cells implicated in abnormal vasculogenesis of gliomas are endothelial progenitor cells (EPCs). These cells are recruited from bone marrow and contribute to tumor vascularization. Strictly, EPCs are not tumoral but stem-like cells with trans-differentiation capacity that contribute to tumors aggressiveness [119].

### Hepatocellular Carcinoma (HCC)

The transcription factor Twist1 is frequently expressed in the nucleus of HCC cells. Up-regulation of Twist1 enhances VM and its knockdown prevents VM formation. Twist1 increases HCC cells plasticity by up-regulation of VE-cadherin and down-regulation of E-cadherin [14]. Another report also suggests that Twist mediates hypoxia-induced VM [48].

Mesenchymal phenotype and poor differentiation in hepatocarcinoma correlates with VM acquisition. No co-relation has been shown between expression of stemness genes and intrinsic VM capacity and it has been proposed that the role of stemness genes in VM capacity of HCC cells is likely to depend on differentiation status [120].

### Non–small cell lung cancer (NSCLC)

In NSCLCs, a Hoechst 33342 dye effluxing side population (SP) cells present features of CSCs. These cells display tubular formation capacity in matrigel [121, 122]. The knockdown of Gli-l (a transcription factor that positively regulates Sox2) by siRNA inhibits VM and stemness capacities of SP cells [123]. YAP1 also regulates the expression of Sox2 by physical interaction with Oct4. NSCLC presents high levels of YAP1 and depletion by knockdown reduces tubules formation capacity in matrigel SP cells [124].

### Breast cancer

Breast cancer stem cells (BCSCs) are identified by CD24-, CD44+ and ALDH+ markers or mammosphere-forming capacity. ALDH+, but not ALDH- cells, have VM formation capacity in matrigel. Epidermal growth factor (EGF) mediates this capacity and can be suppressed by EGFR inhibitor gefitinib or shEGFR. Hsp27 is downstream regulated by EGFR pathway and modulates EGF-mediated VM capacity of BCSCs. Therefore, knockdown of Hsp27 suppresses VM capacity of ALDH+ cells [125]. Ubiquitin-specific protease 44 (UPS44) also contributes to VM formation whereas UPS44 knockdown inhibits VM formation of CSCs in vivo [126].

## Targeting vasculogenic mimicry

As the use of anti-angiogenic therapies against tumor development has had limited results there is a critical need to attempt novel anti-tumor angiogenesis strategies centered in targeting alternative mechanism used by the tumor cells (by way of trans-differentiation) to become pseudo-vascular cells leading to VM.

Numerous studies have attempted to specifically disable VM in different tumor models (Table 3). In a recent report our group has shown that the use of PARP inhibitors was effective in preventing melanoma-derived lung metastasis in a murine model and this effect was in part explained by PARP inhibitor’s faculty to counteract VM through down-regulation of VE-cadherin concomitant to the loss of EMT attributes linked to a decreased vimentin expression and Integrin-Linked Kinase/GSK3β axis down-regulation [127].

**Table 3:** Pharmacological agents targeting VM

Therapeutic agents	Molecular target or function	Effect on VM	References
Bevacizumab (Avastin)	VEGF	no effect	[114]
PARP inhibition	VE-cadherin	inhibition	[127]
Thalidomide (Thalomid)	TNFα; ROS producer	inhibition	[147]
TNP-470 (AGM-1470)	TK inhibition	no effect	[148]
Endostatin (rhEndostatin, Endostar)	integrin signaling	no effect	[149]
Rapamycin (Rapamune)	mTOR, VEGF	inhibition	[150]
Curcumin	EPHA2, PI3K, MMPs	inhibition	[151]
Isoxanthohumol	TGF-β signaling	inhibition	[152]
Vadimezan (ASA404, AS1404, DMXAA)	MAPK, VE-cadherin	inhibition	[140]
Resveratrol	VEGF-R1, VEGF-R2	inhibition	[153]
Ginsenoside Rg3	VE-cadherin/MMPs/EPHA2	inhibition	[154]

The Rho kinase inhibitors fasudil and Incarvin C have also been shown to prevent VM of B16 mouse melanoma cells and HCC in Matrigel and xenograft tumor growth as therapeutic option for targeting cancer VM [128, 129]. An interesting study by Orechia et al. [130] has shown the syndecan-1 co-expression with VM markers in melanoma patient cell lines having vasculogenic/stem-cell like phenotype; melanoma cells lose their ability to form tubule-like structures in vitro after blocking syndecan-1 activity by the specific human recombinant antibody, OC-46 F2 while the combined therapy using OC-46 F2 and L19-IL2, led to a complete inhibition of tumor growth until day 90 from tumor implantation in 71% of treated mice [130].

Another example of VM targeting has used TNBC as proof-of-concept. TNBC cells remaining after conventional chemotherapy readily form VM, which leads to the relapse of cancer after treatment. Functional liposomes vincristine plus dasatinib modified by a targeting molecule DSPE-PEG2000-c(RGDyK), exhibited the superior performances in the enhancement of cellular uptake via targeted action and the induction of apoptosis and removal of VM channels in the TNBC-bearing mice [131].

A similar approach has been reported for VM in glioblastoma multiforme (GBM). Current prognosis of GBM remains extremely poor attributed to the formation of VM and the presence of glioma initiating cells (GICs) responsible for resistence to current therapies and disease recurrence; paclitaxel-loaded liposomes modified with a peptide R8-c(RGD) (R8-c(RGD)-Lip) were used for the treatment of glioma [132]. An in vitro cellular uptake study proved the strongest targeting ability to be that of R8-c(RGD)-Lip to glioma stem cells. Drug loaded R8-c(RGD)-Lip exhibited an efficient anti-proliferation effect on BCSCs and could induce the destruction of VM channels in vitro. The pharmacodynamics study demonstrated that R8-c(RGD)-modified drug-loaded liposomes achieved both anti-VM and anti-BCSC effects in vivo. Finally, no significant cytotoxicity of the blood system or major organs of the drug-loaded liposomes was observed under treatment dosage in the safety evaluation. In conclusion, all of the results proved that R8-c(RGD)-Lip was a safe and efficient anti-glioma drug delivery system [132].

## Conclusion

This review features on one specific strategy that tumor cells utilize to escape from the hostile microenvironment through trans-differentiation leading to a “caricature” of endothelial-like cells, VM. This trans-differentiation allows the tumor mass to evolve to an irrigated, avascular network to avoid shortage of nutrients and oxygen inside the tumor. As we have reviewed, this phenotype change is pertinent to aggressive metastatic behavior and has functional and translational relevance. The molecular pathways underlying VM have lighted up VE-cadherin as a critical component of VM and, therefore it needs to be taken into account for the development of innovative treatment strategies that target tumor cell plasticity and the metastatic properties affiliated with disease recurrence and drug resistance. As a prominent future challenge, new concepts are needed to discern the specific signaling peculiarities of VE-cadherin (different from its role in the endothelial context) affecting tumor cell biology and related to VM/aggressive development. Particular attention is needed to identify the precursors of tumor cells committed to acquire VE-cadherin expression/VM phenotype and to the interactome connecting VE-cadherin with cell trans-differentiation. In this regard, the presence of elevated levels of nuclear VE-cadherin and phospho-VE-cadherin in VM-prone cells (unpublished results) could be crucial to understand the specific role non vascular VE-cadherin in the acquisition of invasive properties. Our ability to effectively abolish cancer is limited in part by the diverse vasculature development in relation with heterogeneous subpopulations contributing to tumors. Moreover, the unintended consequences of hypoxia induced by rapid tumor growth or by some conventional therapies may serve as a catalyst for the VM and CSCs phenotype. Through a profound elucidation of VM biology these limitations could be override. Therefore, it seems prudent, and definitely appropriate, to consider the application of new agents to target VM pathways associated with the stem cell phenotype and resistant to most conventional agents. Targeting VM with specific molecular compounds used in a combinatorial manner with front-line therapies may hold the greatest promise in the war on cancer.
